# Global DNA Methylation of Ischemic Stroke Subtypes

**DOI:** 10.1371/journal.pone.0096543

**Published:** 2014-04-30

**Authors:** Carolina Soriano-Tárraga, Jordi Jiménez-Conde, Eva Giralt-Steinhauer, Marina Mola, Ángel Ois, Ana Rodríguez-Campello, Elisa Cuadrado-Godia, Israel Fernández-Cadenas, Caty Carrera, Joan Montaner, Roberto Elosua, Jaume Roquer, on behalf of GeneStroke, “The Spanish Stroke Genetics Consortium”

**Affiliations:** 1 Department of Neurology, Neurovascular Research Group, IMIM-Hospital del Mar (Institut Hospital del Mar d’Investigacions Mèdiques), Universitat Autonoma de Barcelona/DCEXS-Universitat Pompeu Fabra, Barcelona, Spain; 2 Laboratory of neurovascular pharmacogenomics and genetics, Fundació per la Docència i Recerca Mutua Terrassa, Terrassa (Barcelona), Spain; 3 Neurovascular Research Laboratory, Institut de Recerca, Universitat Autònoma de Barcelona, Hospital Vall d’Hebron, Barcelona, Spain; 4 Cardiovascular Epidemiology and Genetics group, Institut Hospital del Mar d’Investigacions Mèdiques (IMIM), Barcelona, Spain; The Chinese University of Hong Kong, Hong Kong

## Abstract

Ischemic stroke (IS), a heterogeneous multifactorial disorder, is among the leading causes of mortality and long-term disability in the western world. Epidemiological data provides evidence for a genetic component to the disease, but its epigenetic involvement is still largely unknown. Epigenetic mechanisms, such as DNA methylation, change over time and may be associated with aging processes and with modulation of the risk of various pathologies, such as cardiovascular disease and stroke. We analyzed 2 independent cohorts of IS patients. Global DNA methylation was measured by luminometric methylation assay (LUMA) of DNA blood samples. Univariate and multivariate regression analyses were used to assess the methylation differences between the 3 most common IS subtypes, large-artery atherosclerosis (LAA), small-artery disease (SAD), and cardio-aortic embolism (CE). A total of 485 IS patients from 2 independent hospital cohorts (n = 281 and n = 204) were included, distributed across 3 IS subtypes: LAA (78/281, 59/204), SAD (97/281, 53/204), and CE (106/281, 89/204). In univariate analyses, no statistical differences in LUMA levels were observed between the 3 etiologies in either cohort. Multivariate analysis, adjusted by age, sex, hyperlipidemia, and smoking habit, confirmed the lack of differences in methylation levels between the analyzed IS subtypes in both cohorts. Despite differences in pathogenesis, our results showed no global methylation differences between LAA, SAD, and CE subtypes of IS. Further work is required to establish whether the epigenetic mechanism of methylation might play a role in this complex disease.

## Introduction

Ischemic stroke (IS) is a complex disease with outcomes of high mortality and long-term disability. Despite current attention to stroke risk factors and preventive treatment, the number of stroke cases has been rising in recent decades, likely because the aging population has increased as well [1]. Stroke pathogenesis involves a number of different disease processes as well as interactions between environmental, vascular, systemic, genetic, and central nervous system factors. About 80% of strokes are ischemic, as opposed to 20% hemorrhagic. Our current research focuses on ischemic stroke and its most common subtypes: large-artery atherosclerosis (LAA), small-artery disease (SAD), and cardio-aortic embolism (CE) [2], [3].

Substantial evidence from studies in twins, families and animal models points to a genetic risk component [4]–[6] associated with stroke and recent genome-wide association studies have identified new variants associated with IS and specific genetic variants to IS subtypes [7]–[9]. These genetic factors can contribute to conventional risk factors such as hypertension, diabetes, or homocysteine concentrations (which have a known genetic component), and may interact with environmental factors, such as smoking habit, or contribute to triggering an intermediate phenotype, such as atherosclerosis. As a result, they may contribute to stroke latency, infarct size, functional recovery, and outcome. However, stroke risk is not completely explained by these genetic factors [10]. Epidemiological data have provided evidence for a genetic component of the disease, but knowledge about its contribution to IS occurrence and characteristics is limited. There is a need to find new biomarkers for stroke risk, and epigenetic involvement is still largely unknown.

Epigenetics is a promising field of growing interest, both because it may help in the study of complex diseases and because it may generate useful biomarkers. Epigenetic mechanisms, such as DNA methylation, regulate high-order DNA structure and gene expression. DNA methylation is the most widely studied epigenetic modification, and is a marker of genomic DNA because it adds a methyl group to the 5-carbon position of cytosine, in a 5′-CpG-3′ context. This dinucleotide is quite rare in mammalian genomes (∼1%) and clusters in regions known as CpG islands. The methylation of the CpG-island is associated with gene silencing [11].

Global DNA methylation (GDM) has been widely used in epidemiological studies because it is cost-effective, has a high-throughput, and provides quantitative results. Luminometric methylation assay (LUMA) measures levels of 5-^m^C residing in the -CCGG- motif [12], [13]. This motif, which represents 8% of all CpG sites and occurs throughout the genome [14], is used as a proxy marker to estimate GDM.

As global DNA methylation changes over time, an association with aging processes and with modulation in the risk of several pathologies has been suggested [15]–[17]. Aberrant GDM has been associated with atherosclerosis, cancer, hypertension, and coronary heart disease [18]–[21]. It also has been reported that stroke patients show global DNA hypomethylation compared with healthy individuals [19]. However, stroke was analyzed disregarding its etiology, and the relationship between GDM and IS stroke subtypes remains unknown.

In the present study, we analyzed global DNA methylation using LUMA in 3 different ischemic stroke subtypes: large-artery atherosclerosis, small-artery disease, and cardio-aortic embolism. Taking into account the differences in pathogenesis, our hypothesis was that some differences in methylation status would exist.

## Materials and Methods

### Ethics Statement

All aspects of the study were approved by the local institutional review board/institutional ethics committee for each cohort, the Clinical Research Ethics Committee of Parc de Salut Mar and the Ethics Committee of the Vall d’Hebron Hospital, Barcelona. All participants or their approved proxy provided their written informed consent for participation.

### Study Participants

The study included 2 independent prospective cohorts of Caucasian IS patients from 2 hospitals in Barcelona, Spain (n = 281 and n = 204), analyzed retrospectively, and 99 healthy participants. Common inclusion criteria were as follows: (1) first-ever IS, (2) brain imaging with CT or MRI, (3) availability of the clinical data supporting the assigned stroke subtype according to TOAST classification [2], and (4) absence of intracranial hemorrhage, neoplasms, demyelinating and autoimmune diseases, and vasculitides. All patients were assessed and classified by a neurologist. Consenting patients recruited from 2005 to 2012 with a diagnosis of IS fulfilling World Health Organization criteria were considered for inclusion. The subjects were recruited in IMIM-Hospital del Mar (HM cohort) from those enrolled in BasicMar (Ministerio de Sanidad y Consumo, Instituto de Salud Carlos III; FIS No. PI051737), an ongoing prospective registry of Ischemic Stroke [22], and in the Neurovascular Research Group of Hospital Vall d’Hebron (VH cohort).

### Stroke Subtype Classification

Using TOAST criteria, patients were classified into 5 categories: (1) large-artery atherosclerosis (LAA), (2) small-artery disease (SAD), (3) cardio-aortic embolism (CE), (4) stroke of other determined etiology, and (5) stroke of undetermined etiology. Diagnoses were based on clinical features and on data collected by methods such as brain imaging (CT/MRI) and cardiac imaging [2]. Only those individuals classified in LAA, SAD and CE subtype were included in this study.

### Demographic and Vascular Risk Factor Variables

In both cohorts, risk factors were collected in a structured questionnaire, as follows: arterial hypertension (evidence of at least 2 elevated blood pressure measurements, systolic >140 mm Hg or diastolic >90 mm Hg, recorded on different days before stroke onset; a physician’s diagnosis; or use of medication); diabetes (a physician’s diagnosis or use of medication); hyperlipidemia (a physician’s diagnosis, use of medication, serum cholesterol concentration >220 mg/dL, LDL cholesterol >130 mg/dL, or serum triglyceride concentration >150 mg/dl); coronary artery disease (documented history of angina pectoris or myocardial infarction); and atrial fibrillation (documented history or diagnosis during hospitalization). We also recorded age, sex, lymphocyte count, and current smoking habits.

### Peripheral Blood Collection and DNA Extraction

DNA samples were extracted from whole peripheral blood collected in 10 mL EDTA tubes. The Chemagic Magnetic Separation Module I system (Chemagen) was used for DNA isolation at Hospital del Mar and PuregenTM (Gentra Systems) at Hospital Vall d’Hebron. At each site, all DNA extractions were performed at the same time and stored together at −20°C. DNA concentrations were quantified using Picogreen assay and nanodrop technology. The quality of DNA samples was visualized in agarose gels.

### Luminometric Methylation Assay (LUMA)

The assay was carried out as described previously [12]. Genomic DNA (300 ng) was cleaved with *Hpa*II + *Eco*RI or *Msp*I + *Eco*RI (New England Biolabs) in 2 parallel reactions containing 2 µl of Tango buffer (Fermentas) and 5U of each restriction enzyme in a final volume of 20 µl. The reactions were set up in a 96-well format and incubated at 37°C for 4 hours. Then 20 µl annealing buffer (20 mM Tris-acetate, 2 mM Mg-acetate pH 7.6) was added to the cleavage reactions. The original LUMA assay was modified by changing the nucleotide dispensing order to eliminate any background and unspecific digestions of DNA samples as previously described [15]. The samples were placed in a PyroMark Q96 ID System (Qiagen) with the following dispensation order: GTGTCACAGTGT. Percentage of DNA methylation was expressed as [1−(*Hpa*II + *EcoRI* ΣG/ΣT*)/*(*MspI + EcoRI* ΣG/ΣT)]*100.

### Statistical Analysis

We tested the association between LUMA global DNA methylation and IS subtypes. Calculation of sample size for the population analysis was based on results from previous analysis (methylation results and dispersion of the variable), in order to achieve a statistical power of 80%, calculated using GRANMO v7.12. LUMA GDM measurements were expressed as a continuous variable and did not show normal distribution by Kolmogorov-Smirnov test. We first used global DNA methylation as a continuous variable expressed in percentage and tested for univariate associations using Kruskal-Wallis or Mann-Whitney U test statistic for categorical variables and Spearman correlations for continuous data. Global DNA methylation was also categorized into quartiles (Q1–Q4) and *χ^2^* and Kruskal-Wallis test were applied. Variables that differed significantly between IS subtypes in univariate analyses (age, sex, hyperlipidemia, and smoking habit) were included in a multivariate model. Coronary artery disease and atrial fibrillation were not included in the multivariate analyses because they were only present in the CE subtype in HV cohort. We tested for differences in LUMA GDM between IS subtypes by regression models, taking LAA subtype and Q1 of global DNA methylation as reference.

All statistical analyses were performed using SPSS version 18.0. Statistical significance was set at a p-value of 0.05.

## Results

A total of 281 ischemic stroke patients from HM cohort and 204 from HV cohort were studied. For each IS subtype, patients were distributed as follows: HM cohort, LAA n = 78, SAD n = 97, and CE n = 106; HV cohort, LAA n = 59, SAD n = 56 and CE n = 89. The clinical and demographic characteristics of the study population are shown in **Table 1**.

**Table 1:** Baseline Characteristics of the Study Participants.

Variables	HM Cohort n = 281				HV Cohort n = 204			
	LAA = 78	SAD = 97	CE = 106	p	LAA = 59	SAD = 56	CE = 89	p
**Age (mean, SD)**	70.5 (9.6)	68.6 (10.4)	75.8 (8.7)	<0.001	69.3 (11.8)	69.8 (11.6)	75 (14.4)	<0.001
**Sex, female**	23 (29.3%)	29 (29.9)	62 (58.5)	<0.001	21 (35.6)	23 (41.1)	53 (59.6)	0.009
**Diabetes Mellitus**	34 (43.6%)	33 (34)	37 (34.9)	0.36	19 (32.2)	18 (32.7)	25 (28.1)	0.8
**Hyperlipidemia**	46 (59%)	37 (38.1)	45 (42.5)	0.016	15 (25.4)	16 (29.1)	21 (23.6)	0.76
**Hypertension**	52 (66.7%)	65 (67%)	82 (77.4)	0.17	35 (59.3)	27 (49.1)	51 (57.3)	0.50
**Current smoking**	29 (37.7%)	27 (28.1%)	11 (10.6)	<0.001	12 (20.3)	10 (18.2)	12 (13.5)	0.52
**Coronary disease**	13 (17.8)	7 (7.7)	20 (19.8%)	0.05	0	0	8 (9)	0.005
**Atrial fibrillation**	0	0	92 (87%)	<0.001	0	0	45 (51%)	<0.001
**Methylation %**	74	74.6	74.2	0.68	76.4	77	77.4	0.12
**(median,** **IQR)**	(71.8–75.9)	(71.8–76.4)	(71.7–76.2)		(75.5–77.6)	(75.2–77.9)	(76.3–77.9)	
**Methylation** **(n, quartiles)**	80.2–75.9 (19, 24.4%)	30 (30.9%)	31 (29.2%)	0.91	81.3–77.6 (15, 25.4%)	19 (36%)	32 (36%)	0.102
	75.9–73.9 (20, 25.6%)	26 (26.8%)	26 (24.5%)		77.6–76.4 (15, 25.4%)	15 (26.8%)	30 (33.7%)	
	73.9–71.7 (20, 25.6%)	17 (17.5%)	23 (21.7%)		76.4–75.5 (15, 25.4%)	5 (8.9%)	12 (13.5%)	
	71.7–65.5 (19, 24.4%)	97 (24.7%)	26 (24.5%)		75.5–68.6 (14, 23.7%)	17 (30.4%)	15 (16.9%)	

Univariate analysis of LUMA methylation as continuous variable and quartile categories. Ischemic stroke (IS), large-artery atherosclerosis (LAA), small-artery disease (SAD), and cardio-aortic embolism (CE), in HM Cohort and HV Cohort. LAA was taken as reference in methylation quartiles.

In order to replicate the previously published work of Baccarelli et al. [19], we analyzed the GDM differences between 281 ischemic stroke patients and 99 healthy individuals, both from the HM cohort. The demographic characteristics of the controls are shown in Table S1. In univariate analysis, methylation showed significant association to age and hypertension in controls, and significant differences between IS patients and controls, p = 0.008 (Figure S1 and Table S2). In multivariate analyses, adjusted by age and hypertension, hypomethylation was associated to IS patients (p = 0.048; OR = 2.5 (95% CI = 2.3–2.7)).

In univariate analyses (**Table 1**), there were significant differences in age, female sex, coronary artery disease, and atrial fibrillation between IS subtypes in both cohorts. Hyperlipidemia (p = 0.016) and smoking habit (p<0.001) were significant only in the HM cohort. There were no significant differences in GDM between IS subtypes in either cohort, as a continuous variable, or in quartile categories of LUMA methylation.

**Figure 1:**
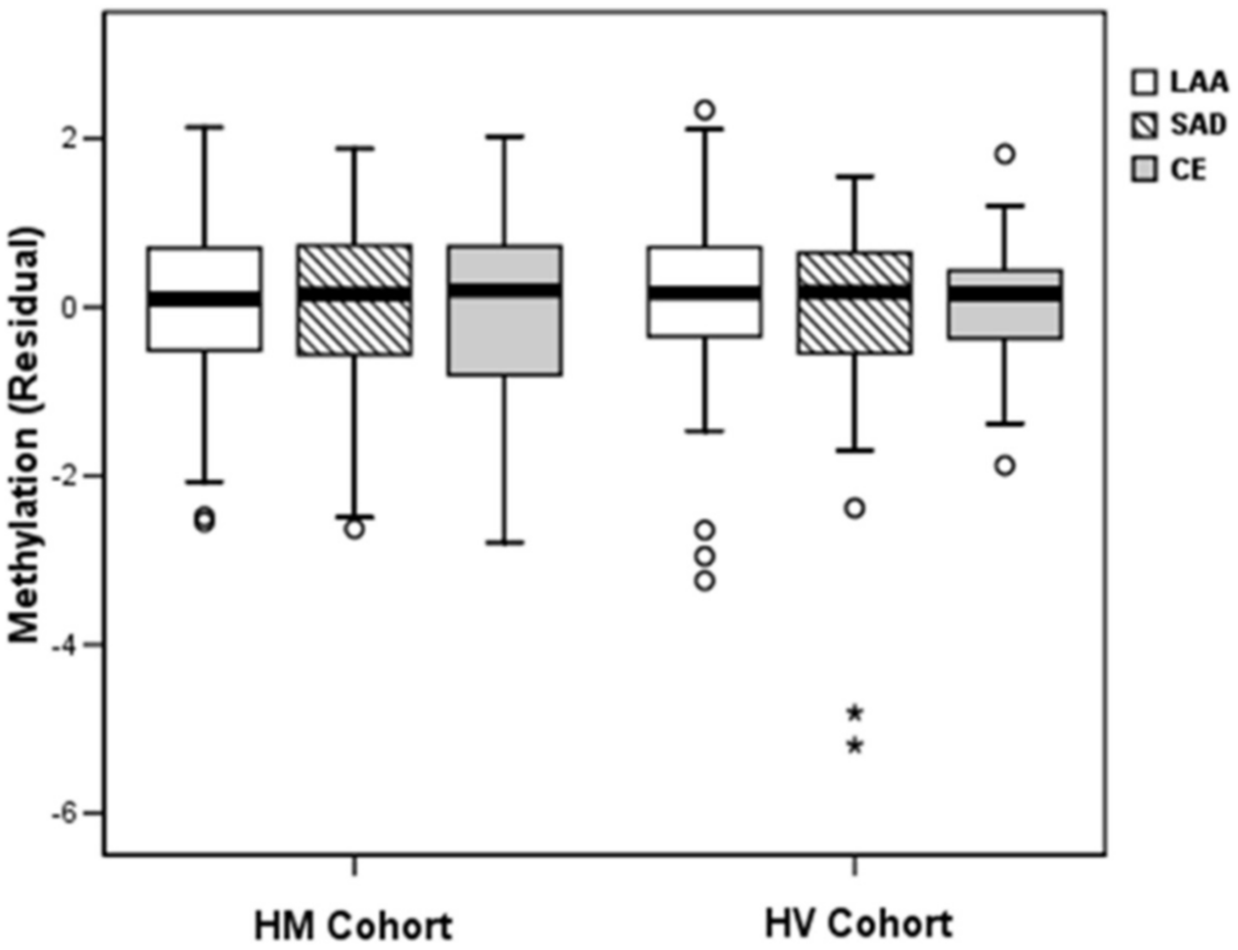
Box plot of global DNA methylation of ischemic stroke subtypes, residuals of multinomial regression. Large-artery atherosclerosis (LAA), small-artery disease (SAD), and cardio-aortic embolism (CE).

In multivariate analyses (**Table 2**), only female sex was independently associated with CE subtype, in both cohorts. Advanced age was associated with CE subtype in HV cohort and showed a statistical trend among HM patients. However, global DNA methylation was not associated with ischemic stroke subtypes in either cohort, neither as a continuous variable nor in quartile categories of LUMA methylation (**Figure 1**). Despite borderline differences in the HV cohort, these were not replicated in the other cohort. Our results were adjusted by the percentage of lymphocytes in all multivariate regression models, with no changes in the final results (data not shown).

**Table 2:** Multivariate analysis for ischemic stroke subtypes.

		HM Cohort							HV Cohort						
		LAA	SAD			CE			LAA	SAD			CE		
		OR	OR	95% CI	p	OR	95% CI	p	OR	OR	95% CI	p	OR	95% CI	p
**Age**		1	0.97	0.94–1.00	0.06	1.03	0.99–1.07	0.08	1	1.00	0.97–1.03	0.98	1.03	1.00–1.06	0.03
**Sex**		1	0.94	0.47–1.89	0.86	0.37	0.19–0.74	0.004	1	0.83	0.37–1.87	0.66	0.42	0.20–0.89	0.02
**HL**		1	0.38	0.20–0.71	0.003	0.46	0.24–0.88	0.02	1	1.17	0.50–2.71	0.72	1.01	0.45–2.26	0.98
**SMK**		1	0.46	0.22–0.96	0.04	0.33	0.14–0.77	0.01	1	0.93	0.33–2.68	0.90	1.38	0.50–3.83	0.53
**Methylation (%)**		1	1.01	0.92–1.12	0.81	1.02	0.92–1.13	0.74	1	0.97	0.83–1.13	0.69	1.17	0.98–1.40	0.08
**Methylation**	**Q4**	1	1.05	0.44–2.51	0.91	1.19	0.49–2.90	0.71	1	0.98	0.36–2.65	0.97	1.76	0.66–4.74	0.26
**(quartiles)**	**Q3**	1	0.86	0.36–2.10	0.74	1.33	0.53–3.31	0.54	1	0.81	0.29–2.28	0.70	1.44	0.53–3.93	0.47
	**Q2**	1	0.71	0.28–1.76	0.46	1.04	0.41–2.61	0.94	1	0.26	0.08–0.92	0.04	0.58	0.19–1.72	0.32
	**Q1**	1	1		.	1		.	1	1		.	1		.

Large-artery atherosclerosis (LAA), small-artery disease (SAD), and cardio-aortic embolism (CE), in HM and HV cohorts. LAA was taken as reference in the multinomial regression. SMK = Smoking habit; OR = odds ratio; p = one-side p value; HL = hyperlipidemia.

Additionally, we performed univariate analysis of methylation as a dependent variable (**Table 3**). Methylation was not associated with IS subtype in either cohort. In the HM cohort, LUMA hypomethylation was associated with diabetes (p = 0.03) and had a nearly significant association with advanced age (p = 0.08), hyperlipidemia (p = 0.06) and CAD (p = 0.08). In the HV cohort, hypomethylation was associated only with male sex (p = 0.03).

**Table 3:** Univariate analysis of LUMA methylation as continuous dependent variable, in HM and HV cohorts.

Variables	HM cohort		HV cohort	
	Methylation (%)	p-values	Methylation (%)	p-values
**ETOAST**	73.9, 74.6, 74.2	0.680	76.4, 76.9, 77.4	0.122
**(LAA, SAD, CE)**				
**Sex (F/M)**	74.5, 74.2	0.538	77.4, 76.8	0.027
**Age (correlation)**	−0.104	0.083	0.046	0.511
**Diabetes Mellitus (Y/N)**	73.7, 74.8	0.034	77.0, 77.0	0.76
**Hyperlipidemia (Y/N)**	73.7, 74.8	0.058	77.0, 77.1	0.513
**Hypertension (Y/N)**	74.2,74.3	0.72	77.0, 77.0	0.706
**Current smoking (Y/N)**	74.2, 74.4	0.894	76.8, 77.1	0.411
**Coronary disease (Y/N)**	73.7, 74.5	0.082	77.6, 77.0	0.363
**Atrial fibrillation (Y/N)**	74.2, 74.5	0.638	77.5, 76.9	0.181

Large-artery atherosclerosis (LAA), small-artery disease (SAD), and cardio-aortic embolism (CE).

## Discussion

Ischemic stroke is a heterogeneous disease with different etiological subtypes as the result of differences in physiopathology and genetic variants [7]. Global DNA hypomethylation of stroke patients has been reported compared with healthy individuals [19] and we replicated, as well. This study is the first to analyze global DNA methylation differences between IS subtypes. Contrary to what might be expected, there were no consistent GDM differences between large-artery atherosclerosis, small-artery disease, and cardio-aortic embolism strokes**.**

Changes in DNA methylation contribute to inter-individual phenotypic variation and are associated with cancer development and other complex diseases [21], [23]. However, there is GDM variability in blood DNA, which has been associated with age, sex, alcohol consumption, white blood cell counts, and method used for DNA methylation measurement [24]–[27]. These variables must be considered both in the statistical analysis and in interpreting the results. Differences in the proportion of white blood cell types and cell type methylation profiles might be a confounder in DNA methylation analysis [25], [27]. For this reason, we adjusted by percentage of lymphocytes but our results were unchanged.

Loss of genomic DNA methylation has been found in a variety of common aging-related diseases, including stroke. Risk of stroke is associated with cardiovascular risk factors and underlying diseases such as atherosclerosis, atrial fibrillation, and hypertension. Atherosclerosis is characterized by global hypomethylation [28], [29], and reports of low genomic methylation have been described in patients with cardiovascular disease [19], [29], [30]. These results enhance the interest in GDM as a potential biomarker of cardiovascular disease risk. However, it has not still been clarified whether GDM is causally linked to cardiovascular disease and atherogenesis.

There are only two previous publications about stroke global methylation, both referring to the same cohort of individuals [19], [31]. Those studies showed that hypomethylation of the repetitive LINE-1 elements analyzed in blood DNA are associated with ischemic heart disease and stroke. However, a limited number of stroke patients were included in these analyses (55 white males), and stroke subtypes were not specified. In our cohort, we replicate their results.

In our study, ischemic stroke cases were classified, according to TOAST criteria, as LAA, SAD, and CE and no significant differences in global DNA methylation were observed using LUMA. Methylation differences in 4 variables (age, male sex, diabetes and hyperlipidemia) showed a trend or a borderline significance in at least one of the cohorts. These results are consistent with previous evidences in global methylation, with different methodologies revealing a decrease in DNA methylation during aging [15], [17], [19], [32].

Our study has a larger sample size than previous studies in the field, and we did not identify GDM differences between IS subtypes. In a post-hoc analysis, we estimated the sample size needed to achieve statistical significance with a small GDM differences. In the most optimistic scenario, assuming the highest difference in global DNA methylation between groups in one of the cohorts as the difference to find (73.6% to 74.0%) with alfa error of 0.05 and a statistical power of 80%, we would require n = 2383 individuals for each subgroup (n = 7149 for each cohort, n = 14298 for the global study). This may have an uncertain clinical and biological significance in a nonspecific epigenetic determination.

Absence of differences between IS subtypes could be explained by detecting, with LUMA, mainly the effect of aging, vascular risk factors, and the baseline diseases of IS patients in the global methylation profile. Although Baccarelli et al. [19] showed that global hypomethylation is already determined in stroke patients and statistically significant compared with healthy individuals, it could result in a similar global hypomethylation between the IS subtypes analyzed in our study. For these reasons, applying new technologies such as genome bisulphate sequencing of specific genome regions and methylation microarrays might help to determine gene-specific methylation patterns underlying GDM and show a distinctive methylation profile between the stroke subtypes.

The limitations of the study are related, first, to its retrospective design. Second, we were unable to adjust for alcohol consumption because of an excess of missing data across one of the cohorts. However, in the other cohort we were able to include alcohol in the analyses but this adjustment did not make any difference.

This study described for the first time that, using LUMA, there is no significant methylation difference between three ischemic stroke subtypes, large-artery atherosclerosis, small-artery disease, and cardio-aortic embolism. Further work is required to establish the role of epigenetic mechanisms in this complex disease. Future studies may benefit from powerful and specific new technologies such as methylation microarrays that determine genome-wide DNA methylation profiles and whole genome bisulphate sequencing.

## Supporting Information

Figure S1 **Box plot of global DNA methylation of controls and ischemic stroke patients from HM cohort.**
(TIF)

Table S1 **Baseline Characteristics of the controls and ischemic stroke patients from HM cohort.** Univariate analysis of LUMA methylation as continuous variable and quartile categories. Ischemic stroke (IS). Controls were taken as reference in methylation quartiles.
(DOCX)

Table S2 **Univariate analysis of LUMA methylation as continuous dependent variable, in HM controls.**
(DOCX)
