# Supplementary material for: Global DNA Methylation of Ischemic Stroke Subtypes
Source: PLoS One. 2014 Apr 30;9(4):e96543. doi: 10.1371/journal.pone.0096543 (PMC4005764; doi:10.1371/journal.pone.0096543)
Supplement: Table S1 — Baseline Characteristics of the controls and ischemic stroke patients from HM cohort. Univariate analysis of LUMA methylation as continuous variable and quartile categories. Ischemic stroke (IS). Controls were taken as reference in methylation quartiles. (DOCX) [file pone.0096543.s002.docx]

**Table S1.**

**Baseline Characteristics of the controls and ischemic stroke patients from HM cohort**. Univariate analysis of LUMA methylation as continuous variable and quartile categories. Ischemic stroke (IS). Controls were taken as reference in methylation quartiles.

| **Variables** | **Controls n=99** | **IS n=281** | **p** |
| --- | --- | --- | --- |
| **Age (mean, SD)** | 66 (19) | 72 (10) | 0.10 |
| **Sex, female** | 51 (52%) | 114 (41%) | 0.06 |
| **Diabetes Mellitus** | 18 (20%) | 104 (37%) | 0.002 |
| **Hyperlipidemia** | 31 (33%) | 128 (46%) | 0.04 |
| **Hypertension** | 47 (50%) | 199 (71%) | <0.001 |
| **Current smoking** | 18 (19%) | 67 (24%) | NS |
| **Coronary disease** | 8 (10%) | 40 (15%) | NS |
| **Atrial fibrillation** | 5 (5%) | 92 (33%) | <0.001 |
| **Methylation %**  **(median, IQR)** | 75.1 (73.5-76.6) | 74.5 (71.8-76.2) | 0.008 |
| **Methylation**  **(quartiles, n)** | 80.0- 76.6 25 (25%)  76.6- 75.1 24 (25%)  75.4- 73.5 25 (25%)  73.5- 65.4 25 (25%) | 51 (18%)  62 (22.1%)  56 (20%)  112(40%) | 0.017 |
