# Supplementary material for: Global DNA Methylation of Ischemic Stroke Subtypes
Source: PLoS One. 2014 Apr 30;9(4):e96543. doi: 10.1371/journal.pone.0096543 (PMC4005764; doi:10.1371/journal.pone.0096543)
Supplement: Table S2 — Univariate analysis of LUMA methylation as continuous dependent variable, in HM controls. (DOCX) [file pone.0096543.s003.docx]

**Table S2.**

**Univariate analysis of LUMA methylation as continuous dependent variable, in HM controls.**

| **Variables** | **HM controls (n=99)** | |
| --- | --- | --- |
|  | **Methylation (%)** | **p-values** |
| **Sex (F/M)** | 74.6, 74.9 | NA |
| **Age (correlation)** | -0.176 | 0.083 |
| **Diabetes Mellitus (Y/N)** | 74.5, 74.7 | NA |
| **Hyperlipidemia (Y/N)** | 74.7, 74.8 | NA |
| **Hypertension (Y/N)** | 74.4, 75.1 | 0.152 |
| **Current smoking (Y/N)** | 75.1, 74.7 | NA |
| **Coronary disease (Y/N)** | 73.7, 74.8 | NA |
| **Atrial fibrillation (Y/N)** | 76.0, 74.7 | NA |
